# Supplementary material for: Powerful turbidity currents driven by dense basal layers
Source: Nat Commun. 2018 Oct 5;9:4114. doi: 10.1038/s41467-018-06254-6 (PMC6173716; doi:10.1038/s41467-018-06254-6)
Supplement: Supplementary file 2 — Description of Additional Supplementary Files [file 41467_2018_6254_MOESM2_ESM.pdf]

Description of Additional Supplementary Files

File Name: Supplementary Data 1
Description: Irreversible pressure/depth changes detected during events.

File Name: Supplementary Data 2
Description: Flow event velocities as measured by BEDs and between instruments

File Name: Supplementary Data 3
Description: AMT temperature and depth vs. time (every 45 minutes)

File Name: Supplementary Data 4
Description: BED and AMT depth vs. time during 24 November 2016 flow event

File Name: Supplementary Data 5
Description: Monterey Canyon thalweg from EM300 data

File Name: Supplementary Data 6
Description: Monterey Bay maximum wave height vs time (every 2 hours) during the experiment

File Name: Supplementary Data 7
Description: Discharge measured at USGS 11152500 SALINAS R NR SPRECKELS CA during the experiment
