# Supplementary material for: Powerful turbidity currents driven by dense basal layers
Source: Nat Commun. 2018 Oct 5;9:4114. doi: 10.1038/s41467-018-06254-6 (PMC6173716; doi:10.1038/s41467-018-06254-6)
Supplement: Supplementary file 10 — Supplementary Data Cover Page [file 41467_2018_6254_MOESM10_ESM.pdf]

**Powerful turbidity currents driven by dense basal layers**

Paull et al.

Supplementary Data 1. Irreversible pressure/depth changes detected during events
Supplementary Data 2. Flow event velocities as measured by BEDs and between instruments
Supplementary Data 3. AMT temperature and depth vs. time
Supplementary Data 4. BED and AMT depth vs. time during 24 November 2016 flow event
Supplementary Data 5. Monterey Canyon Thalweg
Supplementary Data 6. Monterey Bay maximum wave height vs time
Supplementary Data 7. Discharge measured at USGS 11152500 SALINAS R NR SPRECKELS CA during the experiment
